# Paired-Pulse TMS and Fine-Wire Recordings Reveal Short-Interval Intracortical Inhibition and Facilitation of Deep Multifidus Muscle Fascicles

**DOI:** 10.1371/journal.pone.0159391

**Published:** 2016-08-10

**Authors:** Hugo Massé-Alarie, Edith Elgueta Cancino, Cyril Schneider, Paul Hodges

**Affiliations:** 1 Laboratory of Clinical Neuroscience and neuroStimulation, Université Laval (Dept of Rehabilitation), CHU de Québec Research Center, Neuroscience Unit (CHUL), Quebec City, QC, Canada; 2 The University of Queensland, NHMRC Centre of Clinical Research Excellence in Spinal Pain, Injury & Health, School of Health & Rehabilitation Sciences, Brisbane, Qld Australia; University of Sydney, AUSTRALIA

## Abstract

**Objective:**

Paired-pulse transcranial magnetic stimulation (ppTMS) is used to probe inhibitory and excitatory networks within the primary motor cortex (M1). These mechanisms are identified for limb muscles but it is unclear whether they share properties with trunk muscles. The aim was to determine whether it was possible to test the intracortical inhibition and facilitation of the deep multifidus muscle fascicles (DM) and at which inter-stimulus intervals (ISI).

**Methods:**

In ten pain-free individuals, TMS was applied over M1 and motor evoked potentials (MEP) were recorded using fine-wire electrodes in DM. MEPs were conditioned with subthreshold stimuli at ISIs of 1 to 12 ms to test short-interval intracortical inhibition (SICI) and at 15 ms for long-interval intracortical facilitation. Short-interval facilitation (SICF) was tested using 1-ms ISI.

**Results:**

SICI of DM was consistently obtained with ISI of 1-, 3-, 4- and 12-ms. Facilitation of DM MEP was only identified using SICF paradigm.

**Conclusions:**

A similar pattern of MEP modulation with ISI changes for deep trunk and limb muscles implies that M1 networks share some functional properties.

**Significance:**

The ppTMS paradigm presents a potential to determine how M1 inhibitory and excitatory mechanisms participate in brain re-organization in back pain that affects control of trunk muscles.

## Introduction

Coordination of the back muscles is modified in many individuals with chronic low back pain (CLBP) and changes in the functional organisation of the primary motor cortex (M1) may contribute. For example, delayed activation of deep trunk muscles in CLBP [1, 2] was related to a different organisation of the of M1 representation of these muscles [3]. Further, unlike the separate discrete M1 representations of the deep fascicles of multifidus (DM) and superficial erector spinae (ES) muscles in pain-free individuals, LBP involves their convergence/overlap [4] and this is consistent with the more coupled behaviour of these muscles in pain [2]. The mechanisms responsible for these differences remain unknown, but modification of M1 synaptic function is suggested by preliminary data on altered intracortical inhibition and facilitation of corticospinal inputs to the abdominal muscles in CLBP [5]. The techniques used to study these mechanisms are complex and have only begun to be applied to investigation of corticomotor inputs to the trunk muscles. Most work has been undertaken for the limb muscles, but neural mechanisms controlling these muscle groups differ in several respects (e.g. greater bilateral drive to trunk muscles [6]), and it is unclear whether muscles that are controlled primarily for postural tasks share similar cortical mechanisms as limb muscles that are commonly recruited for focal tasks. Intracortical synaptic mechanisms involving trunk muscles might not be inferred from studies of limb muscles.

Intracortical inhibition (ICI) and facilitation (ICF) [7] are probed by paired-pulse transcranial magnetic stimulation (ppTMS) over a single discrete M1 area. Responses to TMS over an M1 area are measured by the motor evoked potentials (MEP) obtained in the muscles. It is generally accepted that ppTMS applied with a short inter-stimulus interval [ISI] (1–5 ms) activates M1 mechanisms of GABA_A_-dependent ICI (short-interval ICI; SICI), i.e. decreases the MEP amplitude, and this inhibition is the greatest for ISIs of 1 and 2.5–3 ms [8–10]. Longer ISIs (6–15 ms) appear to activate mechanisms of M1 facilitation that is argued to be glutamatergic-dependent (see [11]). However, ICF is largely decreased or absent during muscle contraction [12]. Short-interval ICF (1- to 5-ms) is induced with both stimuli near motor threshold [12] (SICF), represents different excitatory circuits that connect directly the corticospinal cell, and can be probed during muscle contraction unlike ICF (6–15 ms) [13, 14]. Although preliminary observations of modified SICI for abdominal muscles in CLBP are promising [5], little is known about the best parameters to study ICI and ICF in trunk muscles and interpretation is based on assumptions coming from studies of limb muscles.

There are several key limitations for investigation of trunk muscle pathways with ppTMS. First, it is difficult to produce large MEP [15]. Second, trunk muscles have complex, multi-layered anatomy, which necessitates intramuscular recordings to accurately represent the individual muscles, which change uniquely in LBP [2, 16]. Available studies that have tested ppTMS paradigms for trunk muscles have used different parameters (precluding systematic examination of effect of each parameter) and non-selective surface recordings (precluding interpretation for individual muscles) [5, 17, 18]

This study aimed to evaluate whether it was possible to obtain ICI and ICF of DM M1 area using ppTMS with selective electromyography (EMG) recordings using intramuscular electrodes. From studies of limb muscles we hypothesized that the greatest SICI of DM M1 area would be induced using ISIs of 1 and 3 ms, whereas greater ICF would be obtained using a SICF paradigm than with ICF.

## Material and Methods

### Participants

Ten pain-free right-handed participants (age: 26±9 years; weight: 63±9 kg; height: 171±8 cm; 6 males) were recruited from a convenience sample for one testing session. Exclusion criteria related to TMS safety guidelines [19] and included, for example, previous brain surgery or lesion, epilepsy or family history of epilepsy, pacemaker/pump holder, medication acting on the central nervous system, and metallic implants in the skull or jaw. People with a history of LBP that was sufficient to compromise function or cause the individual to seek treatment were excluded as decreased SICI has been identified for some muscles [5]. The study and the informed consent signed by the participants were approved by the Institutional Medical Ethics Committee (University of Queensland's Human Research Ethics committee) and all procedures conformed to the Declaration of Helsinki.

### Electromyography (EMG)

Bipolar fine-wire electrodes threaded in a needle (two strands Teflon coated stainless steel wire, 75 μm diameter, 1 mm Teflon removed, tips bent back ∼1 and ∼2 mm to form hooks) were inserted with ultrasound imaging guidance into the right DM at the L4-5 level. A ground electrode was placed over the iliac crest. EMG data were amplified 2000 times, band-pass filtered between 20 and 1000 Hz and sampled at 2 kHz using a Power1401 Data Acquisition System with Spike2 software (Cambridge Electronic Design, Cambridge, UK). The peak-to-peak amplitude of the non-rectified DM MEP was monitored in real-time and the root-mean-square (rms) of rectified EMG and rectified MEP were calculated in real-time on different channels.

### Transcranial magnetic stimulation (TMS)

TMS was applied with the participants positioned in prone lying during right hip extension that was sufficient to activate the DM to 10% maximal voluntary contraction (MVC). MVC was measured during three repetitions of a maximal resisted trunk extension and the peak rms EMG amplitude for 1 s was used to calculate the target of 10% MVC. Hip extension was identified in pilot studies as a straightforward task to control the amplitude of DM EMG and ensure large MEP at minimal stimulator output. The rms EMG amplitude of the right DM was displayed in real-time on a computer screen along with the target at 10% MVC. However, in four participants, 20% MVC of DM was used because no MEP could be detected at 10%. TMS was applied over M1 at a site that elicited the largest MEP in DM muscle using a 70-mm double-cone coil connected to two Magstim 200 monophasic stimulators (BiStim^2^ module, The Magstim Company Limited, Whitland, UK). The center of the coil was first placed 2 cm lateral to the vertex (Cz, 10–20 EEG localization) and placement was adjusted in small increments until highest MEP rms amplitude was identified. This M1 “hotspot” was marked with a pen on the scalp as a reference for consistent coil positioning over the course of each participant’s testing. The coil was oriented to induce current flow in the brain in an antero-posterior (AP) direction. Data from studies of limb muscle suggest that AP currents over M1 are optimal to probe SICI because they activate the late indirect waves of corticospinal volleys, i.e. where the SICI interneuron population preferentially acts [10, 20]. The active motor threshold (AMT) was determined as the TMS intensity that elicited at least five MEPs, which were clearly discernible from background EMG activity, out of 10 trials [21]. This method was used as the conventional 200 μV MEP amplitude used to determine AMT for distal muscles is not possible with paravertebral muscles where only small MEP amplitude (~100 μV at 120% AMT) has been reported in previous studies [15, 21–23].

### Paired-pulses paradigms

Paired-pulse TMS was applied to the left hemisphere with ISIs at 1-ms increments from 1 to 12 ms using a subthreshold conditioning stimulus (CS) at 70% AMT and a supratreshold test stimulus (TS) [7] with sufficient stimulus intensity to produce an MEP of ~500 μV peak-to-peak amplitude. This was achieved with stimulation intensity of 120% AMT in 3 participants, and a higher stimulation (129–159% AMT) was required for 7 participants. Although MEP amplitude of 1 mV is used for studies of hand/forearm muscles, this was not possible for the trunk muscles because of issues including the selective nature of fine-wire EMG recordings and the location of the cortical representation of trunk muscles [17].

For 15-ms ISI, CS was set at 80% AMT which is known to favour facilitation (Chen et al., 1998; Ridding et al., 1995) and TS intensity was monitored as for the 1- to 12-ms ISI. Fifteen unconditioned MEPs and 15 conditioned MEPs were recorded per paradigm. In the SICF paradigm, the TS (set at 100% AMT) was elicited 1 ms before the CS (set at 90% AMT), and 8 MEPs were recorded [12, 24]. The different ppTMS paradigms were randomized to avoid a bias of order. Fifteen unconditioned TS alone were tested at the onset and end of each experiment to monitor any change of corticospinal excitability over the time of experiment.

### Data analysis

**Fig 1:**
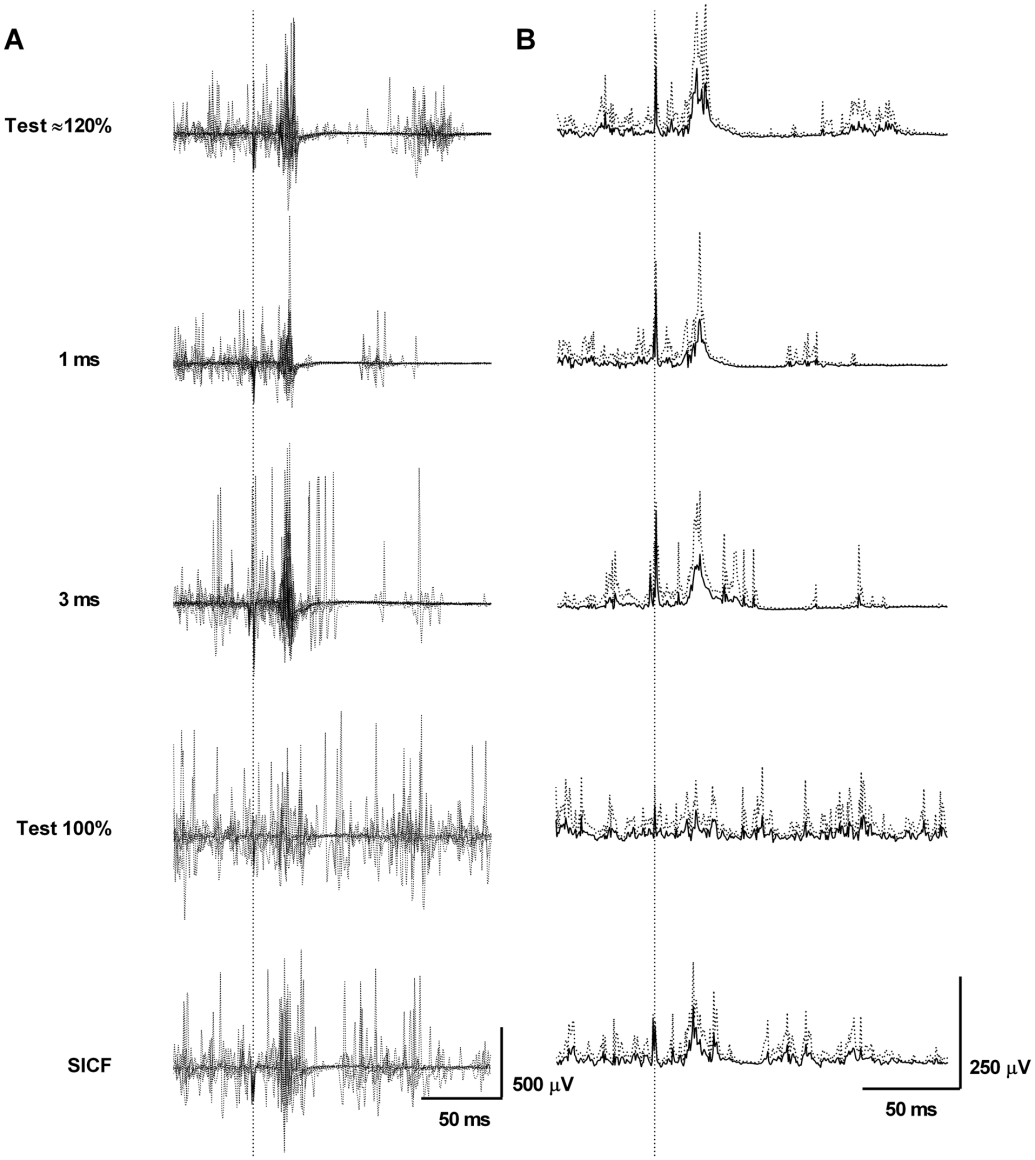
MEP raw and rectified traces. Means (solid lines) ± SD (dotted lines) of MEP traces **A.** From top to down, unconditioned MEP elicited at ~120% AMT and its relative conditioned MEP obtained at 1- and 3-ms inter-stimulus intervals (ISI) and unconditioned MEP elicited at 100% AMT and its relative conditioned MEP obtained with SICF paradigm (1-ms ISI). **B.** Rectified traces equivalent to unrectified of panel A. Note MEP inhibition at 1- and 3-ms ISIs and facilitation for SICF paradigm. MEP: motor evoked potentials; AMT: active motor threshold; SICF: short-interval intracortical facilitation.

EMG was displayed visually for each TMS pulse for visual identification of MEP onset and offset (Fig 1). As the peak-to-peak amplitude of the MEP recorded by fine-wire electrodes varied as a result of inter-trial difference in the motor units excited by the descending volleys, the rms EMG amplitude was calculated between MEP onset and offset [25]. Background rms EMG between -100 to -5 ms before the stimulation was subtracted from the MEP amplitude. The ppTMS MEP amplitude was calculated as the average conditioned MEP rms amplitude expressed as a percentage of the average unconditioned MEP rms amplitude.

### Statistical analysis

A one-way repeated measures ANOVA was used to compare absolute MEP amplitude between Conditioning paradigms (unconditioned vs. conditioned MEP at each ISI; repeated measure). Shapiro-Wilk’s test was performed to determine the normality of the distribution. Since most of the absolute data was not normally distributed and repeated measures analysis was performed on the 14 conditions (Test 120, 1–12, 15 ms), absolute data were transformed with a natural logarithmic function (Ln) and were normally distributed after the transformation (p>0.05). The best paradigms to probe inhibition and facilitation were tested by a one-way ANOVA with repeated measures on traditional inhibitory (1–5 ms) and excitatory paradigms (6–15 and SICF) from hand muscles. As the background EMG (%MVC) differed between participants (in order to induce a measurable MEP), a secondary analysis was undertaken to investigate whether the effect of conditioning was influenced by amplitude of background EMG using a two-way repeated measures ANOVA to compare Conditioning paradigms (conditioned MEP at each ISI; repeated measure) and MVC (10% vs. 20%; between groups factor). A one-way repeated measures ANOVA tested whether the unconditioned MEP changed between the initial trials and those at the end of each experiment to test whether corticospinal excitability was the same throughout the study. Duncan’s multiple range test was used for *post-hoc* testing. The consistency of the effect of ISI on DM MEP amplitudes between participants was determined using the coefficient of variation (CV) and Cohen’s D test. Cohen’s D was calculated by comparison of the unconditioned and conditioned MEP amplitudes at each ISI to determine the effect size. The benchmark for interpretation of the effect size were; small–[0.2–0.5[, medium–[0.5-.8[, and large—≥0.8 [26]. The level of significance was set at p<0.05. Data are presented as mean ± SD throughout the text and figures.

## Results

The mean AMT of the group was 43.5 ± 6.9% of maximal stimulator output and the mean TS intensity to elicit an unconditioned MEP amplitude of ~500 μV (peak-to-peak) was 134.0 ± 12.3% AMT. No difference of amplitude was found between unconditioned MEPs collected at the beginning and the end of experiment (p = 0.10), which indicates that the same unconditioned MEP amplitudes were obtained for the same test TMS intensity and thus the excitability of DM corticomotor pathway remained constant throughout the experiment.

### ISI-dependent modulation of DM MEP

**Fig 2:**
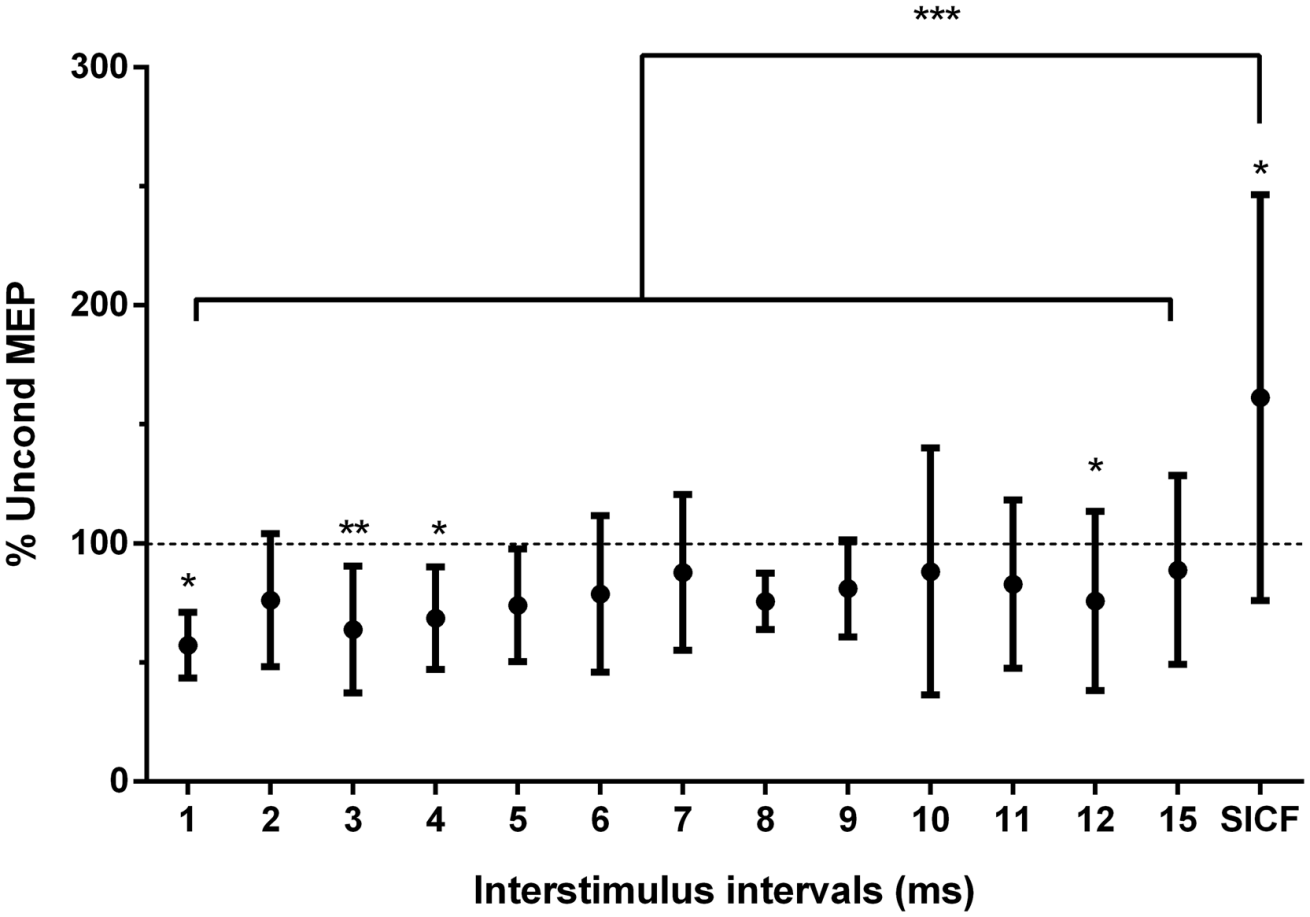
Paired-pulse TMS at interstimulus intervals of 1 to 15 ms and SICF paradigm. MEP are expressed as a percentage of the unconditioned MEP. Note the significant MEP inhibition at 1-, 3-, 4-ms and 12-ms ISIs and facilitation only for SICF paradigm. * p < 0.05. MEP: motor evoked potentials; AMT: active motor threshold; SICF: short-interval intracortical facilitation.

ANOVA applied on raw data detected a main effect of ISIs (F_(13, 117)_ = 1.87; p = 0.04), with a significant decrease of DM conditioned MEP amplitudes, relative to mean unconditioned amplitudes, at ISIs of 1, 3, 4 and 12 ms (*post-hoc analysis* p<0.05, Table 1). Large effect size was reported for 1–5, 8, 9, 11 and 13 ms (Cohen’s D test, Table 1). No other ISI from 1 to 15 ms induced a MEP change that reached significance level although 2 and 5 ms were close to significance (p = 0.07 and p = 0.06 respectively). Fig 2 illustrates the ISI-dependent modulation of the conditioned MEP of DM when expressed in percentage of the unconditioned amplitude.

**Table 1:** Comparison between conditioned and unconditioned MEP for the different paired-pulses paradigms.

Paradigms	Uncond MEP (rms)	Cond MEP (rms)	% Uncond MEP	P*	Effect size*	CV (%)
Test ~120% AMT	0.11 ± 0.09					
1 ms		0.06 ± 0.04	57.3 ± 13.8	***0*.*01***	2.23	24.1
2 ms		0.08 ± 0.07	76.3 ± 28.0	0.07	0.90	36.7
3 ms		0.06 ± 0.05	63.9 ± 26.7	***0*.*003***	1.26	41.9
4 ms		0.07 ± 0.05	68.6 ± 21.6	***0*.*02***	1.27	31.5
5 ms		0.08 ± 0.05	74.1 ± 23.6	0.06	1.15	31.9
6 ms		0.08 ± 0.07	78.9 ± 33.0	0.09	0.67	41.8
7 ms		0.10 ± 0.08	87.4 ± 32.8	0.23	0.45	37.4
8 ms		0.08 ± 0.07	75.8 ± 11.9	0.11	2.03	15.7
9 ms		0.08 ± 0.07	81.2 ± 20.4	0.17	0.95	25.2
10 ms		0.09 ±0.08	88.3 ± 51.9	0.09	0.45	58.8
11 ms		0.11± 0.11	82.9 ± 35.4	0.14	0.90	42.8
12 ms		0.10 ± 0.14	75.9 ± 37.8	***0*.*04***	0.88	49.8
15 ms		0.10 ± 0.11	89.0 ± 39.7	0.24	0.52	44.6
Test ~100% AMT	0.045 ± 0.055					
SICF		0.068 ± 0.079	161.30 ± 85.2	***0*.*047***	0.74	52.8

MEP: Motor evoked potential; Uncond: unconditioned; Cond: conditioned; AMT: active motor threshold; SICF: short-interval intracortical facilitation; CV: coefficient of variation.

p-value: Duncan’s multiple range test Effect size: *Cohen’s D test*

* p-value and effect size were calculated with transform MEP amplitude.

### SICF paradigm (1-ms ISI, TS before CS)

The post-hoc analysis of ANOVA denoted that the amplitude of the conditioned MEP was significantly greater than the unconditioned MEP obtained at 100% AMT (F_(1, 8)_ = 5.31; p = 0.047, Fig 2) and with a medium effect size (Table 1). This confirms that this specific SICF paradigm (TS 100% AMT 1-ms followed by CS 90% AMT) was efficient to probe facilitation of DM M1 circuits.

### Comparison between traditional inhibition and facilitation paradigms

A one-way ANOVA detected a significant main effect for facilitation paradigms (6–15 and SICF; F_(1, 8)_ = 4.69; p<0.001). Post hoc comparisons showed that relative SICF MEP amplitude was significantly larger than the MEP amplitude using any of the traditional ICF paradigms (ISI 6–15ms; p<0.001). A one-way ANOVA did not detect any difference between traditional SICI paradigms (1–5 ms). As a similar decrease in MEP amplitude was present for 1–15 ms paradigms, a one-way repeated measures ANOVA was used to compare all paradigms. A main effect for Paradigms was detected (F_(13, 117)_ = 5.23; p<0.001), which was explained by a larger relative SICF MEP amplitude (% test MEP) compared to all other paradigms (p<0.001; Fig 2).

### Influence of the baseline contraction intensity

**Fig 3:**
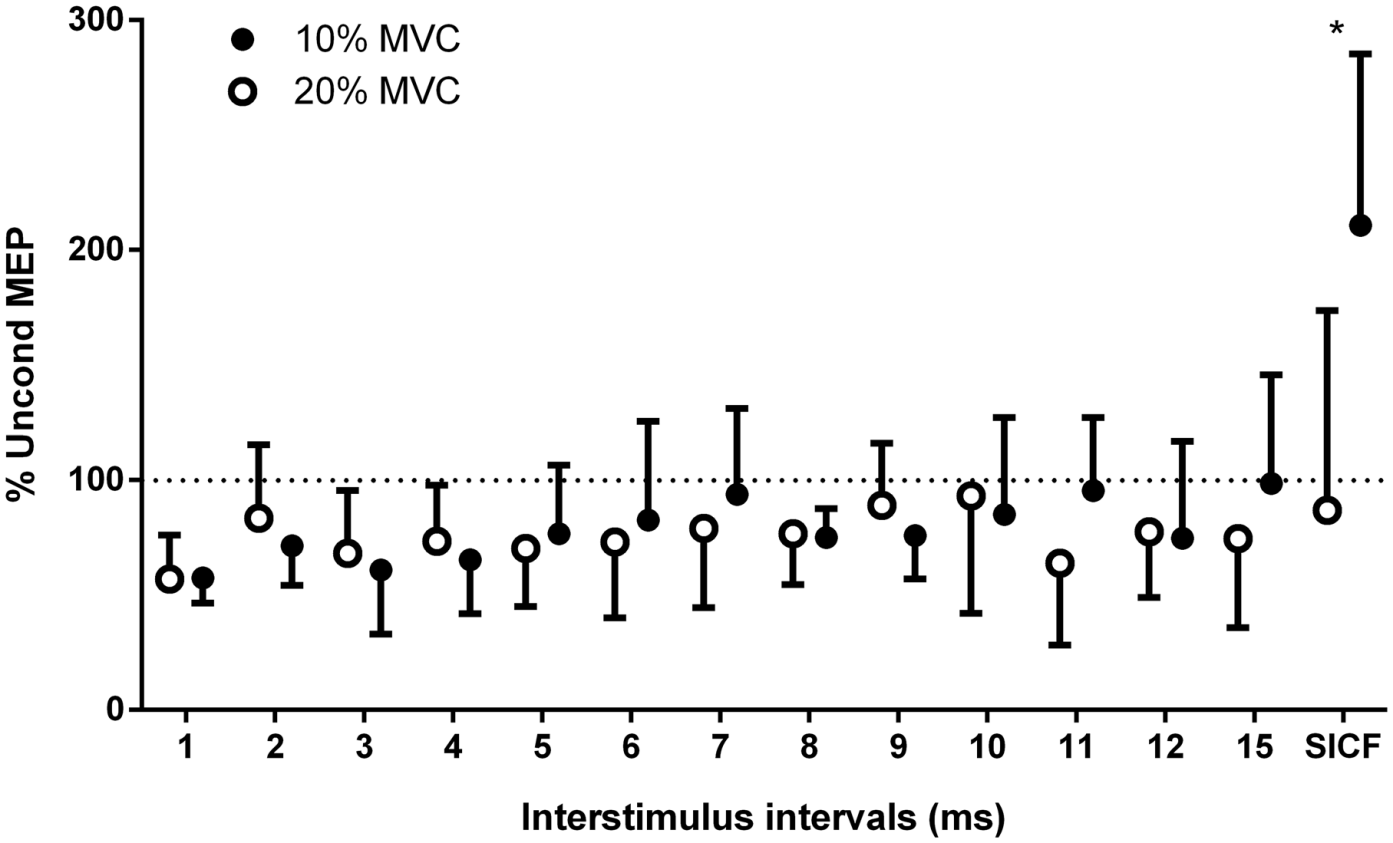
Comparison of paired-pulse TMS data between individuals tested at 10% and those tested at 20% MVC. Note the MEP facilitation for SIFC paradigm. * p < 0.05 between 10% MVC and 20% MVC. MVC: maximal voluntary contraction; MEP: motor evoked potentials; SICF: short-interval intracortical facilitation.

A significant interaction was detected between Conditioning paradigm and Background contraction (%MVC) (F_(1, 13)_ = 3.19; p = 0.0005). *Post hoc* analysis detected that the conditioned MEP was greater for the SICF paradigm when the background EMG was maintained at 10% MVC than at 20% MVC (Fig 3). Background EMG yielded no effect on other conditioning paradigms. CVs were the largest for SICF data and the lowest at 1-ms ISI.

## Discussion

This study tested the mechanisms of M1 inhibition and facilitation of the pre-activated DM muscle. Fine-wire electrodes and ppTMS were used to establish the optimal ISIs optimal to study ICI and ICF in the DM M1 area. Results show that the amplitude of conditioned DM MEP (relative to unconditioned MEP) was significantly reduced at ISIs of 1-, 3-, 4- and 12-ms, and significantly increased using the SICF paradigm only. Consistent with our hypotheses, this concurs with properties of SICI and SICF that have been recorded for limb muscle areas of M1. The effect size for MEP change was large at many ISI (1- to 5-ms, 8-, 9-, 11- and 12-ms) and medium in SICF. Somewhat consistent with our second hypothesis, MEP facilitation was induced with a SICF paradigm (1-ms ISI; CS: 90% AMT; TS: 100% AMT) but not using the ICF paradigm (6- to 15-ms ISI).

Studies that have used ppTMS paradigms to probe ICI and ICF of trunk muscles [5, 17, 18] have used surface EMG recordings that cannot discriminate between activity of different muscle regions. This precludes the interpretation of corticomotor outcomes for overlapping or adjacent muscles. This is important in a clinical context because the control of adjacent muscle fascicles may be modified differently in conditions such as LBP [2]. The results of the present study confirm the feasibility of using fine-wire electrodes to test ICI and SICF of the DM M1 representation using ppTMS paradigms. The underlying mechanisms of ICI and different facilitation paradigms (ICF vs SICF) and their implication in the control of posture and LBP require consideration.

### ppTMS of DM muscle: underlying neural mechanisms

Only one study compare different ISI eliciting inhibition of the MEP [27] and reported a larger effect using 2-ms than 5-ms for the rectus abdominis muscle that concurs with actual results. The level of inhibition of the M1 area controlling superficial multifidus (SM) muscles reported in previous studies [22, 23] range between 77–90% (2-ms ISI) in pain-free participants and 77–120% (2- and 3-ms ISI) in people with CBLP what is less than in the present study (2-ms: 76%; 3-ms: 63%). This discrepancy can be explained by the larger MEP amplitude recorded with fine-wire electrodes and the postural task used (hip extension vs. leaning forward in standing). Indeed, it is possible than a floor effect was reported in previous study because of the small MEP amplitude. Significant inhibition of DM MEPs at 1-, 3- and 4-ms ISIs concurs with studies of limb muscles [28] that show higher levels of SICI at 1 and 2.5–3 ms [8, 9, 29]. In limb muscles, these two intervals are believed to reflect different mechanisms. One hypothesis attributes the 1-ms ISI to the refractory period of the interneurons activated by the preceding CS, thus causing a desynchronization of the descending volleys elicited by the TS applied 1 ms later. In line with this proposal, the amplitude of the first and third I-waves and magnetic D-waves (direct recruitment of corticospinal axons) is decreased following ppTMS with 1-ms ISI [9]. D-waves cannot be elicited by M1 supramaximal stimulation during the 0.7 to 1.1 ms absolute refractory period of the human corticospinal tract, and submaximal stimulation of M1 increases this refractory period up to 1.1 to 2.2 ms [30]. Not all authors agree with the hypothesis of the refractory period for conditioned MEP inhibition at 1-ms ISI [10, 29]. This is because refractoriness predicts less inhibition with higher TS (activation of more excitatory pathways that might overcome the inhibition and the refractory period), which has not been shown. In contrast, the 3- to 5-ms ISIs have been acknowledged to represent activation, by the conditioning TMS, of the inhibitory GABAergic interneuron population, given the substantial inhibition of the late I-waves on which the GABAergic population acts preferentially [8, 9]. Our data suggest that the M1 area of DM has properties of GABAergic mechanisms similar to that of the M1 areas of limb and trunk muscles.

No study has tested ICF for paravertebral muscles in pain-free participants. Only one study reported ICF (15 ms) of SM in people with CLBP and did not report any facilitation of the MEP test [23]. Analysis of facilitation mechanisms is likely to be complicated by baseline EMG activity as these circuits might be involved in voluntary contraction, which could saturate the interneurons. This may explain the general decrease of the conditioned DM MEP amplitude at ISIs > 5 ms (all below 100%, significant inhibition at 12-ms ISI and large effect size at 8-, 9-, 11- and 12-ms—Fig 2 and Table 1). An alternative explanation could be the direction of the current used for TMS. AP current in the brain reduces the conditioned MEP amplitude at ISIs >5 ms (6–15 ms) because of the inhibition of late I-wave even at these ISIs, but not when using a postero-anterior (PA) brain current [13, 20, 28]. Hanajima et al. (1998) reported that inhibition of the MEP test occurred up to 20-ms ISI (1- to 20- ms ISI) when AP current is used [31]. This implies an influence of current direction on recruitment of specific subpopulations of interneurons [20, 32]. ICF involves different intracortical mechanisms than SICI [33] as evidenced by the greater CS intensity required to produce ICF [17, 32] and the different response to drug treatments [34]. Thus, the intensity of the CS used (70% AMT) to probe ICF requires consideration. We cannot exclude the possibility that ICF may have been elicited with higher CS intensity [17]. Comparison of ICF with ppTMS at rest would help clarify these issues, but this is not possible as DM MEPs were highly variable and infrequently observed in that state.

Only two studies have probed SICF of SM, although with surface EMG electrodes [22, 23]. The present results are similar with significant SICF of DM MEP amplitude. In general studies on hand muscles have tested SICF using the protocol of Ziemann et al. (1998) which involves a TS at ~1 mV (first stimulus) followed by a CS at 90% MT [14]. This protocol produced series of peaks and troughs with excitatory ISI at 1.1–1.5 ms, 2.3–2.9 ms and 4.1–4.4 ms when the hand muscles are at rest. During muscle contraction, only one peak is present between 1.1–1.5 ms [14]. By extrapolation from studies of hand muscles, a 1-ms ISI might not have been the optimum ISI to probe SICF. Future studies could test multiple ISI and stimulation intensities for the TS and CS to characterize SICF profile of DM. Despite this consideration, the SICF paradigm used was the only paradigm that produced significant facilitation of DM MEP amplitude and this is consistent with studies of hand and trunk muscles [22, 23]. The SICF paradigm produces facilitation in pre-activated muscles [12, 13] and increases the early and late I-waves of the descending volleys [33, 35, 36] and represent a different interneurons population than ICF [33]. SICF populations are thought to involve excitatory glutamatergic-dependant cortico-cortical interneurons synapsing on the corticospinal cells [24, 36]. MEP facilitation with the SICF paradigm was observed for DM pre-activation at 10% MVC but not at 20% MVC (Fig 3). This concurs with TMS studies of limb muscles [13, 35]. It was suggested that a higher volitional contraction that engages a larger M1 area for more recruitment of corticospinal cells and motor units may have pre-facilitated the I-wave circuits’ interneurons, thus rendering them less available for recruitment by ppTMS and hindering M1 facilitation. Group variability of SICF data (at 10% and 20% MVC) might be explained by the fact that different neural systems not probed by ppTMS (e.g. sub-cortical, brainstem, and propriospinal projections in addition to ipsilateral and contralateral corticospinal drives [37]) can control DM, and their respective contribution to DM pre-activation using the leg elevation task could vary between participants.

Muscle activation was used to obtain a MEP of sufficient amplitude to be conditioned since it is difficult to produce MEP for paravertebral muscles *at rest* (small or no MEP amplitude [15]). However, muscle contraction reduces the capacity to condition the MEP and study inhibition or facilitation [20, 28]. In a pre-activated muscle, a larger pool of spinal motoneurons will be closer to depolarization threshold than at rest. As a result, motoneurons might have been more easily activated by the early I-waves of the descending volleys. This could have decreased the contribution of late I-waves [20] under the inhibitory control of SICI interneurons population [38]. This might have reduced the capacity to probe larger M1 inhibition of DM [20, 28], as already evidenced in limb muscles [10, 20, 39]. Our study is the first to test multiple ISIs for deep back muscles and the findings are consistent with studies that have used similar parameters to elicit ICI and ICF for hand and trunk muscles [7, 12, 28]. Further studies that probe the properties of intracortical mechanisms controlling deep trunk muscles should vary the stimulation intensity at each ISI, the current direction, the level of muscle pre-activation and use single-unit recording and pharmacology combined with TMS.

### Implication for control of deep trunk muscles

Our results on SICI and SICF that probe the DM M1 area with similar parameters as those for limb muscles suggest that the control of posture and trunk muscles may include contribution from M1 circuits and shares some properties with the control of limb movement.

Although we did not test the implication of SICI/ICF in the control of DM, previous studies on M1 area controlling hand muscles have shown that intracortical mechanisms are involved in motor planning and coordination [33]. For instance, the modulation of the level of SICI during specific hand/finger tasks has revealed that M1 must be released from GABAergic inhibition for motor programming [40], during specific phases of one-joint movement [41] and during proximal-to-distal joint synergies [42]. In contrast, GABA levels in M1 must be increased before muscle relaxation [43]. One possible implication is that M1 circuits could be involved in the control of deep trunk muscles (e.g. DM which provide fine-tuning of intervertebral movement [44]). TMS studies have already demonstrated representations of trunk muscles at M1, with discrete areas for adjacent paravertebral muscles such as deep and superficial back muscles [45] and the link between M1 organisation and the latency of their anticipatory postural adjustments [46]. One recent study also denoted that greater ability to volitionally activate DM relies on higher transsynaptic excitability of the DM area of M1 [22]. These studies and our findings suggest that the control of limb and trunk muscles might share some similar circuits and mechanisms of M1. Further studies should test directly whether SICI/ICF circuits are involved in the fine-tuning of trunk muscles since this knowledge can impact the understanding of the physiopathology of low back pain.

### Implication for low back pain

Recent works showed that SICI was absent in the M1 representation of the abdominal and SM in chronic LBP and this was accompanied by impaired motor control [5, 22]. Altered motor control during movement could contribute to the recurrence of pain by repetition of microtrauma [2, 16]. A release of M1 from GABAergic inhibition may also contribute, over time, to the loss of discrete boundaries between M1 areas controlling adjacent muscles, and thus explaining the overlap of cortical representation of separate back muscles such as deep and superficial back muscles, and the loss of their individual control in LBP. These mechanisms and their link with the control of posture in pain-free individuals and those with LBP require further investigation of M1 circuits using ppTMS and fine-wire electrodes recordings of multiple deep muscle fascicles in combination with functional tasks that demand anticipatory postural adjustments.

### Methodological considerations

The between-subject variability of MEP modulation was high at some ISIs and very low at others. This implies the variation was not explained by the recording methods. In addition to physiological mechanisms already targeted for SICF, variability of DM data could be due to individual differences in coordination of superficial and deep back muscles to perform the hip extension task used to control pre-activation of the muscle during TMS testing [47]. Using a TS at AMT could induce more variable SICF than a TS at 120–130% AMT [48] and might explain the variability we observed for this paradigm. As SICF variability was lower when data were grouped separately for participants who activated DM to 10% and 20% MVC, further studies should investigate the importance of the contraction level and TS MEP amplitude on SICF variability [12, 14]. Finally, TMS-generated AP currents chosen here to selectively recruit the late I-waves where intracortical circuits of M1 preferentially act could have also activated the supplementary motor and premotor areas [49], thus providing another source of variation to TMS outcomes of DM muscle.

### Conclusions

The present study tested the potential for ppTMS paradigms and fine-wire EMG recordings to explore the intracortical motor circuits involved in the motor control of DM fascicles. ISIs of 1, 3, 4 and 12 ms were optimal to probe SICI of M1 area, and only the SICF paradigm (test TMS at 100% AMT elicited 1 ms before conditioning at 90% AMT) induced facilitation in the pre-activated muscle. Our findings suggest that M1 circuits controlling DM, limb and superficial trunk muscles share similar properties. These results are of importance to better understand the control of deep trunk muscles and its impairment in conditions such as LBP.

## Abbreviations

M1: primary motor cortex
MEP: motor evoked potential
ICI: intracortical inhibition
ICF: intracortical facilitation
ISI: interstimuli interval
TMS: transcranial magnetic stimulation
DM: deep multifidus
